# Some Effects of Immigration from a Dental View

**Published:** 1900-06

**Authors:** Frank R. Dickerman

**Affiliations:** Taunton, Mass.


### SOME EFFECTS OF IMMIGRATION FROM A DENTAL VIEW.[1]

BY FRANK R. DICKERMAN, D.M.D., TAUNTON, MASS.

At intervals we may see government statistics of the Bureau of Immigration, or extracts of them published in various forms; and we may well wonder that this country assimilates the new arrivals so readily and with so little trouble. If we are very much interested in such matters, we may even go to the trouble of hunting up literature on the subject; and we find an immense amount of rather dry reading; or, to put it another way, the interesting portion is such a small part of the whole, and is separated from the mass only with such great difficulty, that we drop the subject, saying to ourselves, "We pay taxes for some one to look after just such things as these; guess we'll let them earn their salaries."

Perhaps nearing election time we learn which candidate will have the votes of the Italio-American, Franco-American, or the German American Campaign Club, or a riot of striking coal-miners or some sweat-shop investigations again call our attention to the subject and we do more reading; but more commonly we learn through some one else that the population is more largely composd of foreign-born men and women, but just how many does not concern us very much. Not to burden you with figures, let us

---

[1] Read before the Harvard Odontological Society, September 28, 1899.

take Boston as a typical large city of the eastern district of the country. We find that in 1895 the percentage of foreign born to the total population was about thirty-six per cent.; those of foreign parentage to total population, about seventy-one per cent. Such a percentage would hardly prevail outside of the large cities except in certain localities almost exclusively settled by a certain class. These localities generally call for some special class of labor, and the products of this class of labor form almost the entire bulk of production of that locality. In a measure, these comers from other lands may be divided into two classes,—those who become citizens after a due lapse of time, and are probably in the majority, and those who do not care for the rights of citizenship for various reasons.

It has been stated that in proportion as these new-comers become citizens so much the more readily do they become assimilated; the racial lines are broken and set aside and a general broadening of that people occurs. Intermarriage of this class with outsiders, if we may call them such, is more common than with those who do not become citizens, always excepting those who are closely bound by some religious faith and by customs subservient to that faith. It seems to me that if one could follow out a line in this breaking down of racial distinction on the one hand, and the drawing closer and firmer on the other, some very interesting features would come to light.

Students have presented to us their views on heredity as concerns crime, pauperism, insanity, and various other matters, and it would seem that the laws that govern these things would also control the physical characteristics of a people. These characteristics would of course include the physical development of the jaws and teeth.

Those of us who are in the smaller cities and towns and who do more of a general practice than our city brethren may often note that the teeth of those who are foreign born and who have attained middle age or more in this country, present widely varying features. This difference ranges from one or two pin-head cavities in an otherwise almost perfect set of teeth to a most complete wreck of what was the same almost perfect set of teeth ten years, or even less, before. These teeth when good are usually short, thick, and of a yellow tint, the bicuspids and molars are short, heavily covered with enamel, and nearly square in shape. The third molar is fully developed, and occludes well with the upper molar. However, a very small cavity in these teeth causes much trouble from the extreme sensitiveness. Where one or two teeth are missing from such a set of teeth, the left inferior first molar is more apt to be the favorite; if another should be missing, the chances are more than good that it would be on the right side,—more often the upper second bicuspid or the first molar.

Then we have the same kind of teeth almost entirely destroyed by black or brown decay, attrition, and crumbling. In other cases the teeth are lost wholly from pyorrhœa alveolaris. This trouble generally winds up the career of the teeth of this class. Then we see those who have indifferently good teeth in their native country; cases where the white horn-like decay has reduced the teeth to a choice collection of roots and abscesses; this results from a five years' or less residence in this country, no sickness of importance or other contributing cause being known. This statement is upon the patients' authority, so there may be some slight contributing cause after all, but it is quite doubtful. In these cases which I have cited the employment, habits, manner of living, and pretty nearly the same diet were about the same as that of their native country.

Right here I would say that I do not wish to convey the idea that before these people came to this country they did not suffer with toothache and other kindred ailments, that all immigrants have good or even fair teeth, or that in their native country there were no bad teeth. People in those countries do have toothache and do have bad teeth,—in some cases, notoriously bad teeth. In order to watch the deterioration of a tooth, we ought to have a tooth to start with and to watch. Therefore I have made no account of the collections of roots and abscesses with which some of these people burden themselves.

Foreign-born children of foreign parentage coming to this country seem to suffer for a time more severely than their parents, but in the end apparently come out as well if not a little better. Coming here at the age when they are about to cut the permanent teeth, the temporary teeth suffer quite badly, crumbling away until not much is left but the roots. These roots are left in many times, and quietly go about their work of keeping space for the permanent teeth or favoring irregularities. The permanent set does not seem quite so good as that of the father, but still may be a good set of teeth. Possibly there may be required half a dozen fillings to put them in good order, and the fillings, if properly done, remain in place for a long time.

Later the lower third molar will probably be lost either from crowding or bad texture. Such teeth are quite liable to decay, but there will be a large amount of decay, comparatively speaking, before discomfort is felt, and these teeth may remain in use several years longer than those of their parents.

American-born children of foreign parents are especial sufferers with the early decay of the temporary set. The teeth crumble and wear away to the gum line, and alveolar abscess steps in on the slightest provocation. Then, by forced premature extraction, crowding and irregularities may result. The lower first molars are not of much account in these cases, and are lost anywhere from the seventh to the tenth year, or just about early enough to let the second molars into the space, giving a fair grinding surface. Later the upper first molars and bicuspids may be lost.

A comparison of these two classes just mentioned—foreign-born children of foreign parentage and American-born children of foreign parentage—would show hardly any difference on the whole. If one suffers most at one time, the other seems to be in as much trouble later; so it is almost an even thing.

As a fitting illustration of the three types before mentioned, I will describe three sets of teeth, all of which I have seen within a year and a half. Their possessors also represent three generations,—grandfather, son, and granddaughter. The grandfather is a native of the Western Islands, about sixty-five years old, a carpenter by trade, and came to this country about twenty-eight years ago. He still has a complete set of teeth, in which I was able to find two small cavities only. In these cavities decay had evidently stopped some time ago. These cavities cause no discomfort. The teeth are quite well worn, and the gums have receded somewhat. An upper lateral was broken by an accident. With these exceptions, the teeth might be called perfect.

The son is about thirty-seven years old, carpenter by trade, and came to this country when about sixteen, to escape conscription for the military service required. His teeth seem to be about as good as the father's, but the bicuspids and molars are not quite so large. He has four fillings, mostly in the molars. The left lower first molar is missing.

The granddaughter is about eleven, and what few teeth of the temporary set that are now left are about as bad as can be imagined. Those teeth of the permanent set which have come through are fairly good, strong teeth. Three of the first molars have small fillings in them. Just how these teeth will eventually turn out is, of course, wholly problematical, but now they bid fair to last her as long as she has any need of them, providing a reasonable amount of care be expended on them.

In a locality where a certain race of foreign-born people collect and settle permanently, because their special kind of labor is called for in that locality, we may find a type of jaws, teeth, and facial characteristics transmitted from generation to generation. These people seldom marry outside of their race, and in many cases keep to the customs and habits of their native land, unless the police and health officers interfere. These customs and habits have a direct influence on the mental and physical welfare of such people.

Again, in a locality where the population has a lesser percentage of foreign-born parents, but still a respectable percentage of the whole, we shall still expect to find a certain type of jaws and teeth and other characteristics peculiar to that people. In their descendants are found that same type, or traces of that type modified more or less by intermarriage and environment. In the one case the type tends to be perpetuated; in the other it seems to merge into another distinct type. So this merging-into-something-else process may go on until at some time in the far future there will be found a certain composite, which some very learned person will designate as the American jaw, the American teeth; perhaps he will go farther and add the American face.

Possibly this American face will have something of the "white man's burden" in it, possibly not. That will remain to be seen.

